# Multiparametric MRI analysis for the evaluation of renal function in patients with hyperuricemia: a preliminary study

**DOI:** 10.1186/s12880-021-00675-4

**Published:** 2021-09-28

**Authors:** You-Zhen Feng, Xiang-Nan Dong, Qi-Ting Lin, Ping-Kang Chen, Xiao-Qing Xiong, SiTu DingKun, Long Qian, Zhong-Yuan Cheng, Xiang-Ran Cai

**Affiliations:** 1 Medical Imaging Center, First Affiliated Hospital of Jinan University, No.613 West Huangpu Avenue, Tianhe District, Guangzhou, 510630 Guangdong China; 2 Department of Nephrology, First Affiliated Hospital of Jinan University, Guangzhou, Guangdong China; 3 MR Research, GE Healthcare, Beijing, China

**Keywords:** Hyperuricemia, Kidney, FMRI, IVIM, DTI, BOLD, Multiparametric

## Abstract

**Background:**

To investigate the renal dysfunction in patients with hyperuricemia by employing a multiparametric MRI protocol, consisting of quantitative water molecule diffusion, microstructure, microscopic perfusion, and oxygenation measurements in kidneys.

**Materials and methods:**

A total of 48 patients with hyperuricemia (HU) and 22 age-matched healthy control subjects (HC) were enrolled in the study. For each participant, three different functional magnetic resonance imaging (fMRI) sequences were acquired and analyzed, including intravoxel incoherent motion imaging (IVIM), diffusion tensor imaging (DTI), and blood-oxygen-level-dependent MRI (BOLD). Thereafter, an independent two-sample *t*-test was applied to discover the significant differences of MRI indices between the hyperuricemia (HU) and HC groups, and the specific potential biomarkers between two subgroups of HU group (asymptomatic hyperuricemia group (AH) and gouty arthritis group (GA)). Further, multivariate logistic regression analyses were performed to classify the AH from the GA group using the MRI indices with significant between-group differences. The receiver operating characteristic (ROC) curve was plotted, and the area under the ROC curve (AUC) was calculated to assess the performance of each MR index for differentiation between the AH and GA groups.

**Results:**

Ten parametric values of the HU group were significantly lower than those of the HC group among the 14 fMRI parameters (*P *< 0.05). The cortical D, D^*^, and *f* values and medullary D and R2^*^values had significant differences between the AH and GA groups (*P *< 0.05). Combining the cortical D and *f* values and medullary R2* value gave the best diagnostic efficacy, yielding an AUC, sensitivity, and specificity of 0.967 ± 0.022, 91.67%, and 95.83%, respectively.

**Conclusions:**

A multiparametric MR analysis plays an important role in the evaluation of renal dysfunction in hyperuricemia from multiple perspectives. It could be a promising method for noninvasive detection and identification of the early-stage renal damage induced by hyperuricemia.

**Supplementary Information:**

The online version contains supplementary material available at 10.1186/s12880-021-00675-4.

## Introduction

Hyperuricemia is a condition in which the serum uric acid (SUA) is paranormal (> 7 mg/dL in men or > 6 mg/dL in women). With the rapid increase in the prevalence of hyperuricemia worldwide [1], it has become one of the public health concerns in recent years. Hyperuricemia is an independent risk factor for renal function decline because two-thirds of human urate is excreted through the kidneys. On the other hand, it might potentially be associated with the development and progression of chronic kidney disease (CKD) where urate-lowering therapy (ULT) slows its progression. However, the efficacy of ULT is not uniform across the CKD spectrum. Recent clinical studies reported that those patients with CKD stages 2 and 3 displayed the greatest benefit [2], whereas those patients in CKD stage 4 have no improvement in the eGFR after ULT treatment [3]. Unfortunately, many CKD patients are unaware of their condition because CKD is a silent disease at its early stage. Thus, an early screening of renal dysfunction is urgent for individual patients with hyperuricemia to acquire the greatest beneficial effect of ULT treatment.

The estimated glomerular filtration rate (eGFR) calculated from serum creatinine concentration is known as the standard clinical assessment of renal function. However, it cannot detect the renal damage at an early timepoint [4]. Magnetic resonance imaging (MRI), acting as a noninvasive tool, has been performed to assess the renal structure alterations in routine clinical practice. With the improving hardware and software, it can detect the changes in renal microenvironment and microstructure as well and quantitatively analyze these changes in a single scan session. And many studies suggested that the functional MRI (fMRI) may be a potential optimal candidate tool for the surveillance of kidney function, including obstructive nephropathy [5], diabetic nephropathy [6, 7], and CKD [8, 9].

Intravoxel incoherent motion imaging (IVIM), diffusion tensor imaging (DTI), and blood-oxygen-level-dependent (BOLD) techniques are the most common fMRI tools in assessing the renal function from the hemodynamics (diffusion and perfusion), microstructure (diffusion and directivity of diffusion), and oxygenation perspectives, respectively [10]. Our team has applied the IVIM technique to predict renal dysfunction at the early stage of diabetic nephropathy [11] and hyperuricemia [12]. We found that IVIM parameters might be a sensitive predictor of early diabetic and hyperuricemic nephropathies. Also, we employed a combination of BOLD imaging and DTI to detect early-stage renal fluctuations induced by diabete [13] and and hyperuricemia [14]. However, our previous study mainly focused on the value of IVIM technique or BOLD imaging and DTI in detecting the early renal alterations in patients with hyperuricemia. A prior study [15] employed the multiparametric renal MRI protocol, including phase contrast (PC) MRI, arterial spin labeling (ASL), T1 mapping, IVIM, and BOLD imaging, to assess the renal changes in structure, hemodynamics, and oxygenation in CKD. It suggested that the use of complementary MRI techniques could provide a wealth of information on the pathophysiological mechanisms in CKD. However, whether the combination of the different parameters could improve the diagnostic performance of CKD is still unclear.

In this study, three different quantitative MRI approaches, including IVIM, DTI, and BOLD, were employed to investigate the renal function in patients with persistent hyperuricemia condition in comparison with the healthy controls from multiple perspectives. In addition, gout arthritis and asymptomatic hyperuricemia are currently the two common states of hyperuricemia in clinical practice. Therefore, the optimal parameters were probed to detect the differences between patients with asymptomatic hyperuricemia and patients with gouty arthritis.

## Materials and methods

**Fig. 1:**
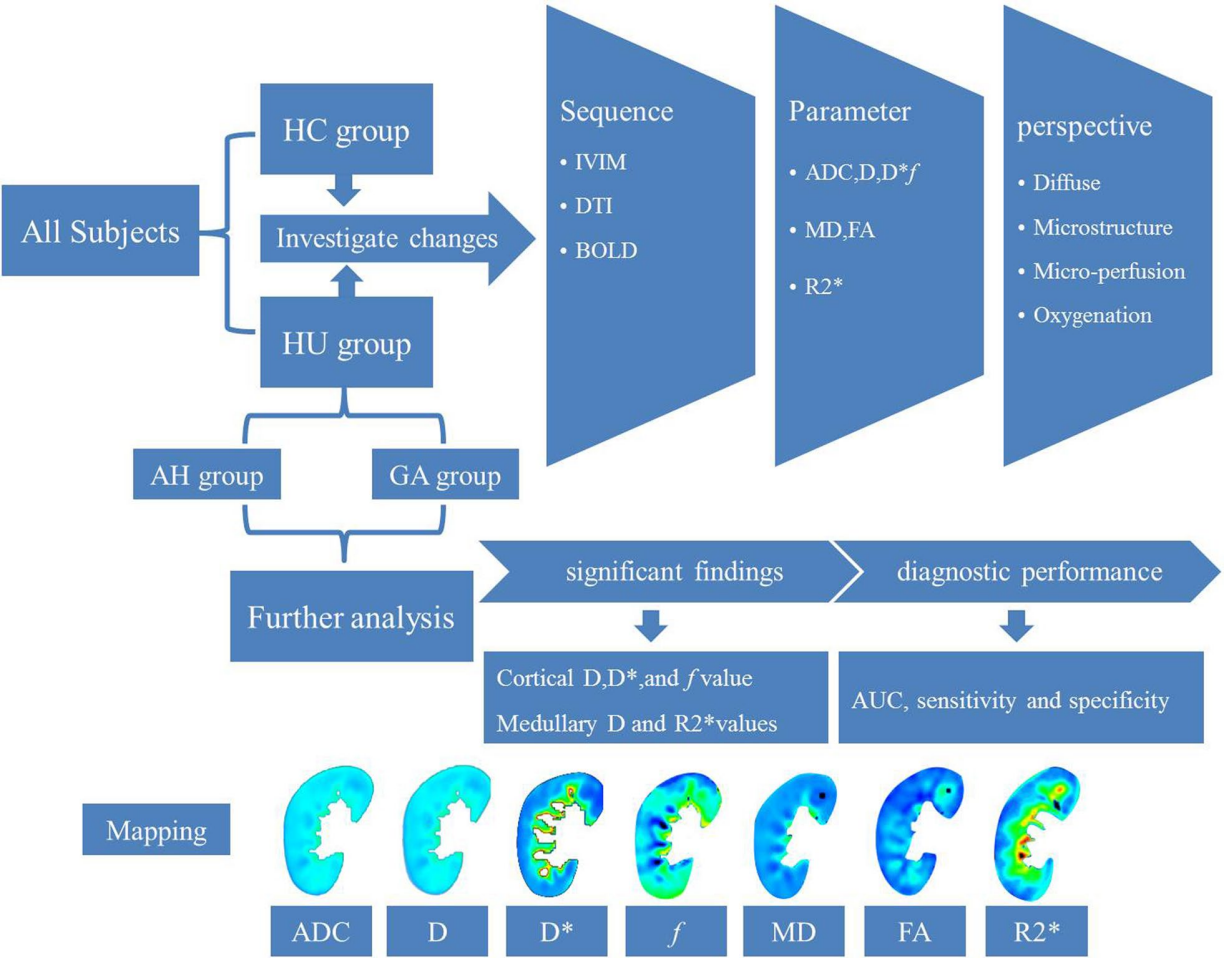
The flowcharts of the current study. HC, healthy control; HU, hyperuricemia group; AH, asymptomatic hyperuricemia group; GA, gouty arthritis group. The asymptomatic hyperuricemia group (AH) and gouty arthritis group (GA) are the subgroups of the HU group

This study was approved by the ethics committee of our institution, and informed consent was obtained from all participants. The outlines of this study were presented in Fig. 1.

### Participants

A total of 79 consecutive participants were enrolled from March 2016 to June 2017. The following inclusion criteria were applied: (1) participants should be males because hyperuricemia and gout predominantly occur in males due to the effects of estrogen; (2) they had no history of kidney diseases, including kidney tumors, glomerulonephritis, and chronic nephritis; (3) they had no hypertension, diabetes, hyperthyroidism, and any known disease potentially involving the kidneys in the past; (4) they should be without any ULT before; (5) they underwent multiparametric MRI of IVIM, DTI, BOLD, and conventional T1-weighted and T2-weighted imaging.

All the participants were divided into two groups, abided by the following criteria: (1) healthy control (HC): SUA levels < 416 µmol/L (7.0 mg/dL) with normal renal function in the past three months; (2) hyperuricemia group (HU): SUA levels > 416 µmol/L (7.0 mg/dL). Furthermore, the HU group was split into two subgroups according to the presence or absence of clinical symptoms, namely, asymptomatic hyperuricemia group (AH) and gouty arthritis group (GA). The patients with GA underwent the first attack of gout which was confirmed clinically complying with 2015 gout classification criteria [16]. The serum creatinine and SUA of all participants were tested the day before the MRI examination. Then, the eGFR was acquired by the Chronic Kidney Disease Epidemiology Collaboration formula [17].

There were nine subjects excluded from this study for various reasons: inadequate renal function examination information (n = 3), multiple renal cysts (n = 2), multiple calculi (n = 2), or poor MRI image quality due to severe motion artifacts (n = 2). Finally, seventy subjects were enrolled in this study, including 22 healthy controls and 48 patients with hyperuricemia (24 subjects with AH and 24 cases with GA).

### The multiparametric MRI protocol

The recruited participants underwent MR scans using a 3.0-T MRI system (MR750, GE Healthcare, WI, USA) with an eight-channel body array coil. A belt-type sensor was wrapped around the abdomen to detect the motion of the abdominal wall. All MR images were obtained in coronal planes (about 11–13 slices). In consideration of the relatively long scan time and the effect of motion artifacts, each participant was given breathing uniformity training before scanning, and breathing trigger mode was employed for IVIM and DTI examination. In addition, all subjects were given breath-holding exercises as well to match the needs of the BOLD scanning.

### Mapping ADC, D, D*, and *f* from IVIM model

IVIM sequence consisted of 11 different b values in total (b = 0, 30, 50, 80, 100, 150, 200, 300, 500, 800, and 1000 mm^2^), incorporated by six lower b values (b < 200 s/mm^2^) and five higher b values (b ≥ 200 s/mm^2^). The other detailed parameters were as follows: SE/EPI sequence; TR/TE, about 8571.4/52.3 ms; matrix size, 128 × 160; bandwidth, 250 kHz; field of view, 38 × 30.8 cm^2^; slice thickness/gap, 4/0.4 mm; and the acquisition time, about 4 min. IVIM-derived parameter was calculated by Eqs. (1) and (2) which were described by Le Bihan et al. [18].

1 $${\text{S}}_{{\text{b}}} /S_{0} = \exp \left( { - {\text{bADC}}} \right),$$

2 $$\frac{{{\text{S}}_{{\text{b}}} }}{{S_{0} }} = \left( {1 - f} \right) \times \exp \left( { - {\text{bD}}} \right) + f \times {\text{exp}}\left( { - {\text{bD}}^{{\text{*}}} } \right).$$

S_b_/S_0_ stands for the signal intensity corresponding to the diffusion gradient b or 0 values. In the IVIM model, D is the pure diffusion coefficient, D^*^ represents the perfusion-related diffusion, and *f* is the pseudo-diffusion fraction.

### Mapping MD and FA from DTI model

DTI images employed 16 directions of diffusion gradient orientation and two different b values (0 s/mm^2^ and 600 s/mm^2^), using SE/EPI sequence: TR/TE, about 7750/56.0 ms; matrix size, 128 × 160; bandwidth, 250 kHz; field of view, 38 × 30.4 cm^2^; slice thickness/gap, 4/0.4 mm; and the acquisition time, about 3 min. Maps of mean diffusivity (MD) and fractional anisotropy (FA) were generated by Eqs. (3) and (4). The value of the eigenvalues (λ_1_, λ_2_, and λ_3_) represents the magnitude of diffusion along the principal axes.

3 $$\text{M}\text{D}=\frac{1}{3}({\lambda}_{1}+{\lambda}_{2}+{\lambda}_{3}),$$

4 $$\text{FA}=\sqrt{\frac{3}{2}}\times \sqrt{\frac{{\left({\lambda}_{1}-\text{M}\text{D}\right)}^{2}+{{(\lambda}_{2}-\text{M}\text{D})}^{2}+{{(\lambda}_{3}-\text{M}\text{D})}^{2}}{{(\lambda}_{1}^{2}+{\lambda}_{2}^{2}+{\lambda}_{3}^{2})}}).$$

### BOLD MRI to map R2*

For BOLD imaging, participants were instructed to hold their breath during acquisition intervals (three times, 15 s each). And the scanning method was implemented with a modified multiecho data image combination sequence (MEDIC) in inspiration and eight T2*-weighted images for each slice. The detailed scanning parameters were as follows: 8 echoes; TR/TE, 150/3.5–28.2 ms; matrix size, 256 × 192; bandwidth, 50 kHz; field of view, 40.0 × 30.4 cm^2^; slice thickness/gap, 4/0.4 mm; flip angle 30°; and the acquisition time, about 45 s. BOLD MRI data used weighted echo time (TE) fit to form T2*/R2* maps from the log of the exponential signal decay (Eqs. 5 and 6). The R2* value can be performed to assess local tissue oxygenation; a low R2* value indicates high oxygenation.

5 $$\frac{{\text{S}}_{\text{T}\text{E}}}{{\text{S}}_{0}}=\text{exp}\left(-\frac{\text{T}\text{E}}{{\text{T}2}^{*}}\right),$$

6 $${ \text{R}2}^{*}=1/{\text{T}2}^{*}.$$

### T1- and T2-weighted imaging

The T1-weighted imaging used breath-hold dual-echo sequence (TR/TE, 3.8/2.2 ms; slice thickness/gap, 4.0/0.4 mm; field of view 38.0 × 38.0 cm; bandwidth, 166.7 kHz; matrix, 256 × 200; number of excitations, 0.7 and scan time, 6 s). The T2-weighted imaging adopted respiratory triggering and fat-saturated fast-recovery fast spin echo (TR/TE, 6000/75.2 ms; slice thickness/gap, 4.0/0.4 mm; field of view, 38.0 × 38.0 cm; bandwidth, 83.3 kHz; matrix, 320 × 320; NEX, 2.5, scan time was about 1 min 48 s).

The total acquisition time of all MR scanning was diverse which mainly depends on the breathing rate of the subject, and the average time was 10 min. Ultimately, seven sets of mappings were obtained from the renal cortex and medulla, including ADC, D, D*, *f*, MD, FA, and R2* mappings (Fig. 1 and Additional file 1: Fig. S1). And a total of 14 sets of mappings were performed in the analysis in this study.

### Data analysis of multiparametric MRI

Both morphological MR images of T1WI and T2WI and the functional MR sequences (IVIM, DTI, and BOLD) were reviewed in consensus by two radiologists (C. Z. Y. six years and F. Y. Z. seven years of experience with MRI, respectively). The radiologists were blinded to the clinical data and grouping information. The eligible functional MR datasets were transferred to a workstation (Advantage Workstation software 4.5; GE Healthcare) for processing to obtain the corresponding mapping.

### The measurement of multiparametric MRI

Considering the following three main reasons, the measurements from the right kidney were selected for the analysis in the current study. Firstly, hyperuricemia is a systemic disease, which involves the bilateral kidney equally; secondly, some previous studies has suggested that there was no statistical difference in IVIM, DTI, and BDLD parameters between the right and left kidneys; finally, the right kidney is less affected by motion artifacts than the left kidney.

All of ROIs were manually sketched on the original image at the median plane or the adjacent median slice of the kidney. Those ROIs were automatically matched to the functional maps to obtain the corresponding quantitative value. Arc-shaped ROI was used to cover the whole cortical area. The ROIs of renal medulla were circular, separately positioned in the upper, middle, and lower poles of the kidney. The ROI size of the renal cortex or medulla was 600–800mm^2^ or 28–45 mm^2^, respectively (Additional file 1: Fig. S2).

### The inter reader agreement assessment

The repeatability of fMRI measurement data was evaluated by interclass correlation coefficients (ICCs). The ICC value was deemed as good consistency if it was greater than 0.800.

### Statistical methods

SPSS Statistics 22.0 (IBM, USA) was employed for statistical analysis. The results of the analysis were exhibited with mean ± standard deviation. The statistical difference was judged by the *P* values less than 0.05. The Independent-Sample Test was adopted to evaluate any significant differences both between the HC and HU groups and between AH and GA groups. The binary logistic regression analysis was used to investigate the effective function for the diagnosis using the combined MR indices in differentiation between AH and GA groups. Afterward, the receiver operating characteristic curve (ROC) was plotted, the area under the ROC curve (AUC) was calculated, and the optimal cutoff values were determined.

## Results

Table 1 presented the results of the comparative analyses of the basic clinical data. The SUA level in the HU group was significantly higher than that in the HC group (*P *< 0.001). Furthermore, most of the patients (AH group (21/24) and GA group (20/24)) had UA in the supersaturated condition (SUA > 480 µmol/L). The eGFR of HU groups was significantly lower than that of the HC group (*P* = 0.001).

**Table 1:** The basic information of participants

	Group		Subgroup of HU		t/*p*	
	HC(*n* = 22)	HU(*n* = 48)	AH (n = 24)	GA (n = 24)	t_1_/***p***_1_	t_2_**/*p***_2_
Mean age (year)	32.32 ± 6.05	32.69 ± 6.13	32.46 ± 6.63	32.92 ± 5.72	− 0.235/0.815	− 0.257/0.799
Age range (year)	24–44	24–44	24–43	22–44	–	–
Mean SUA (µmol/L)	343.57 ± 27.68	591.94 ± 98.63	566.25 ± 76.67	617.64 ± 112.36	− 16.117/< 0.001	− 1.851/0.071
SUA > 480 µmol/L	0	41/48	21/24	20/24	–	–
eGFR (mL/min /1.73 m^2^)	112.72 ± 17.51	95.49 ± 19.11	95.91 ± 20.94	95.08 ± 17.54	3.591/0.001	0.149/0.882

SUA, serum uric acid; eGFR, estimated glomerular filtration rate. HC, healthy control; HU, hyperuricemia group. AH, asymptomatic hyperuricemia group; GA, gouty arthritis group. t_1_/*p*_1,_comparison between the HC and HU groups; t_2_/*p*_2,_comparison between the AH and GA groups; *p* < 0.05 was considered significant

### The results of inter reader agreement assessment

The results of the ICCs were displayed in the Additional file 1: Table S1. The ICCs ranged from 0.846 to 0.971, suggesting good agreements for the measurement data.

### The comparison of multiparameter between the groups

**Fig. 2:**
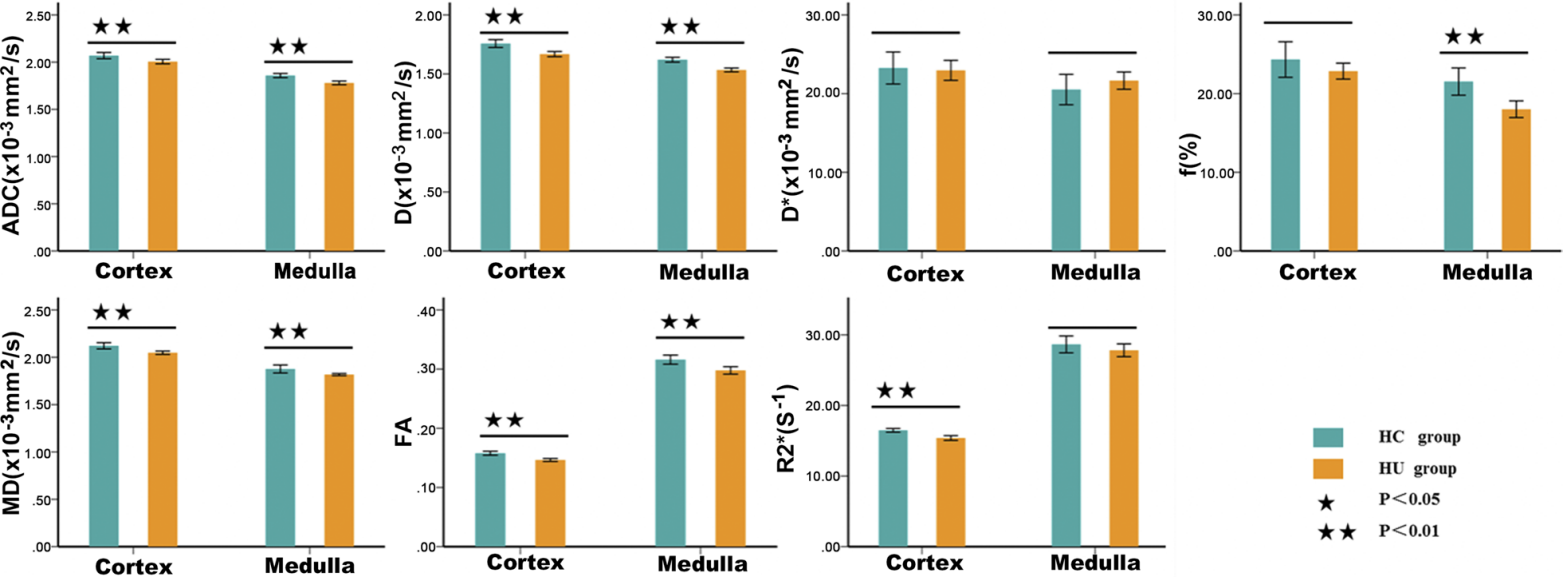
The comparisons of the multiple parameters between the HC and HU groups. HC, healthy control; HU, hyperuricemia group

**Fig. 3:**
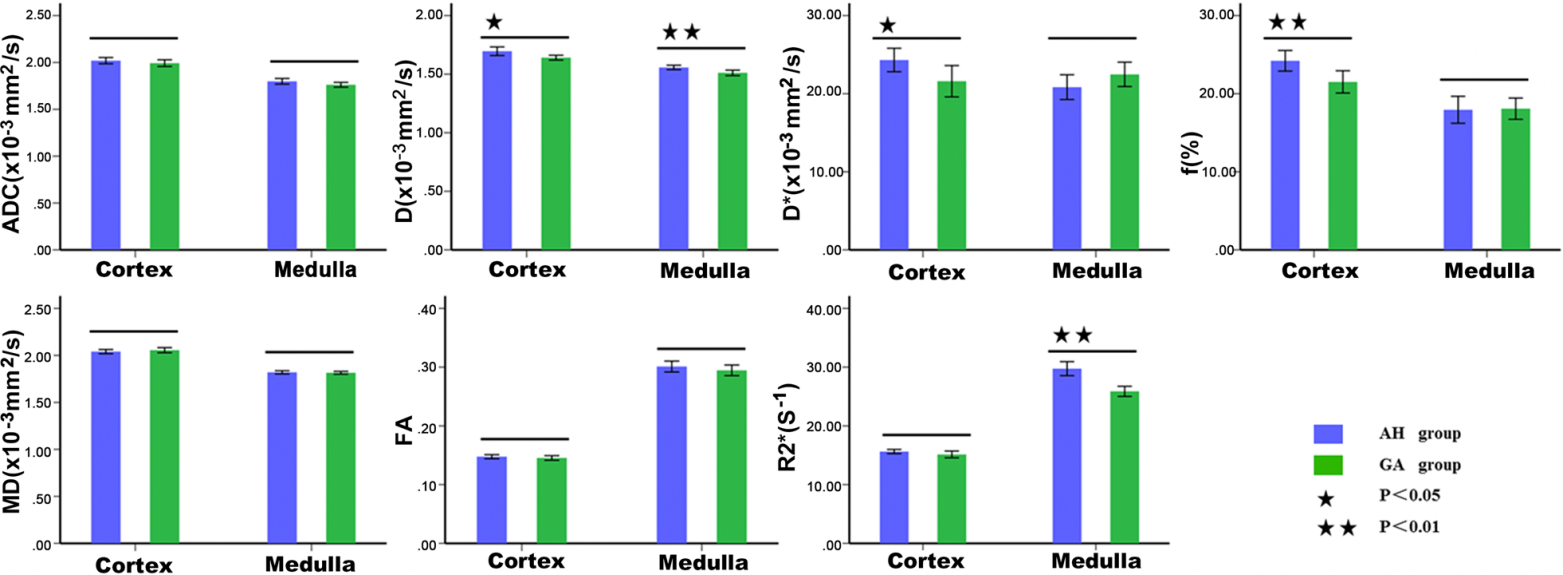
The analysis results of the multiple parameters in the subgroups of the HU group composed by AH and GA groups. AH, asymptomatic hyperuricemia group; GA, gouty arthritis group. The AH and GA groups are the subgroups of the HU group

**Fig. 4:**
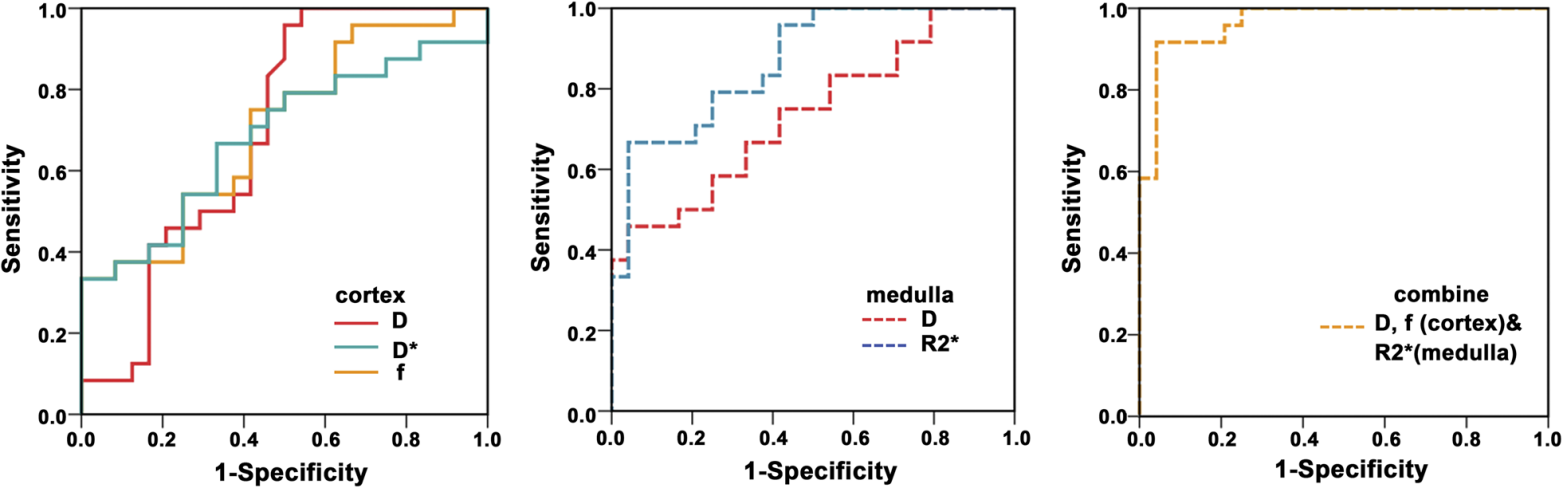
The diagnostic performance of the fMRI parameters for further differentiating between the subgroups of the HU group

The fMRI parametric comparisons between the HC and HU groups were shown in Fig. 2; Table 2. Among the 14 parameters, a total of 10 values of the HC group were significantly higher than those of the HU group (*P *< 0.05). Furthermore, the comparison between the AH and GA groups showed that there were significant differences in cortical D, D^*^, and *f* values and medullary D and R2^*^ values (*P *< 0.05) as shown in Fig. 3; Table 3. The diagnostic performances of these five values were detailedly demonstrated in Fig. 4; Table 4. The R2* value of renal medulla showed the highest AUC value compared to the other values performed in this study. Compared with other values, the D value of the renal cortex had the best sensitivity and worst specificity (95.83% and 50.00%, respectively); the most outstanding specificity was discovered in renal medulla R2* value with poor sensitivity (95.83% and 66.67%, respectively). Combining the cortical D and *f* values and medullary R2* value further improved the diagnostic efficacy, yielding an AUC of 0.967 ± 0.022, a sensitivity of 91.67%, and a specificity of 95.83%.

**Table 2:** The comparison of multiple parameters between HC and HU groups

Method		Cortex		Medulla		t/*p*	
Sequence	Parameter	HC(*n* = 22)	HU(*n* = 48)	HC(*n* = 22)	HU(*n* = 48)	t_1_**/***p*_1_	t_2_**/***p*_2_
IVIM	ADC	2.070 ± 0.074	2.006 ± 0.082	1.858 ± 0.049	1.780 ± 0.068	3.121/0.003	4.837/< 0.001
	D	1.758 ± 0.075	1.668 ± 0.075	1.620 ± 0.046	1.534 ± 0.055	4.624/< 0.001	6.411/< 0.001
	D*	23.255 ± 4.587	22.954 ± 4.377	20.515 ± 4.358	21.653 ± 3.778	0.264/0.793	− 1.115/0.269
	*f*	24.335 ± 5.092	22.859 ± 3.494	21.533 ± 3.904	18.010 ± 3.644	1.233/0.227	3.673/< 0.001
DTI	MD	2.121 ± 0.073	2.048 ± 0.059	1.877 ± 0.095	1.818 ± 0.038	4.469/< 0.001	2.832/0.009
	FA	0.158 ± 0.007	0.146 ± 0.009	0.316 ± 0.017	0.298 ± 0.022	5.329/< 0.001	3.454/0.001
BOLD	R2*	16.478 ± 0.584	15.386 ± 1.150	28.651 ± 2.693	27.815 ± 3.122	5.261/< 0.001	1.083/0.283

HC, healthy control; HU, hyperuricemia group. ADC,D,D* and MD value are given as × 10^− 3^ mm^2^/s; The *f* and FA value are dimensionless; R2* value is given as s^− 1^; t_1_/*p*_1,_the comparison of cortex between the HC and HU groups; t_2_/*p*_2,_ the comparison of medulla between the HC and HU groups; *p* < 0.05 was considered significant

**Table 3:** The comparison of multiple parameters between the subgroups of the HU group

Method		Cortex		Medulla		t/*p*	
Sequence	Parameter	AH(*n* = 24)	GA(*n* = 24)	AH(*n* = 24)	GA(*n* = 24)	t_1_**/***p*_**1**_	t_2_**/***p*_**2**_
IVIM	ADC	2.019 ± 0.079	1.993 ± 0.085	1.798 ± 0.071	1.762 ± 0.061	1.113/0.272	1.914/0.062
	D	1.696 ± 0.087	1.641 ± 0.050	1.557 ± 0.044	1.511 ± 0.056	2.703/0.010	3.171/0.003
	D*	24.311 ± 3.564	21.597 ± 4.754	20.833 ± 3.747	22.472 ± 3.704	2.237/0.030	− 1.524/0.134
	*f*	24.210 ± 3.124	21.508 ± 3.373	17.943 ± 4.095	18.077 ± 3.217	2.878/0.006	− 1.127/0.900
DTI	MD	2.040 ± 0.053	2.055 ± 0.065	1.820 ± 0.041	1.815 ± 0.035	− 0.875/0.386	0.455/0.652
	FA	0.148 ± 0.008	0.145 ± 0.009	0.301 ± 0.022	0.295 ± 0.021	0.878/0.385	1.023/0.311
BOLD	R2*	15.629 ± 0.854	15.144 ± 1.361	29.745 ± 2.827	25.886 ± 2.039	1.478/0.148	5.422/ < 0.001

AH, asymptomatic hyperuricemia group; GA, gouty arthritis group. The AH and GA groups are the subgroups of the HU group. The ADC,D,D* and MD value are given as × 10^–3^ mm^2^/s; The *f* and FA value are dimensionless; R2* value is given as s^−1^; t_1_/*p*_1,_the comparison of cortex between the AH and GA groups; t_2_/*p*_2,_ the comparison of medulla between the AH and GA groups; *p* < 0.05 was considered significant

**Table 4:** ROC analyses in differentiating between AH and GA groups

	MR index	AUC	95% CI	*P* value	Sensitivity (%)	Specificity (%)	Optimal cutoff
Cortex	D	0.697 ± 0.080	0.770,0.966	0.014	95.83	50.00	1.71
	D*	0.684 ± 0.079	0.543,0.854	0.020	70.83	58.33	23.28
	*f*	0.705 ± 0.076	0.556,0.828	0.007	75.00	58.33	19.92
Medulla	D	0.736 ± 0.072	0.531,0.837	0.001	58.33	75.00	1.53
	R2^*^	0.868 ± 0.050	0.596,0.876	< 0.001	66.67	95.83	26.24
Combined	D, *f* (cortex) &R2^*^ (medulla)	0.967 ± 0.022	0.870,0.997	< 0.001	91.67	95.83	–

AH, asymptomatic hyperuricemia group; GA, gouty arthritis group; AUC, area under the receiver operating characteristic curve; CI, confidence intervals

Note that the optimal cutoff values of combining D, *f* (cortex), and R2^*^(medulla) were not provided due to their dependence on other imaging parameters

## Discussion

In the current study, the multiparametric MR analysis of kidneys was performed to distinguish those patients with high uric acid levels from healthy subjects with normal uric acid levels and detect the differences of renal function between asymptomatic hyperuricemia and gouty arthritis. The longitudinal multiparametric MRI measurements consisted of quantitative assessments of ADC, D, D*, *f*, MD, FA, and R2* values. Our results demonstrated that the ADC, D, MD, FA, and R2* values of renal cortex and ADC, D, *f*, MD, and FA values of the renal medulla in patients with hyperuricemia were significantly lower than those in healthy controls. Also, the significant differences in cortical D, D^*^, and *f* values and medullary D and R2^*^values were found between asymptomatic hyperuricemia and gouty arthritis. These findings suggested that the multiparameter combination can not only reveal the alterations of renal function from the various perspectives but also enhance the sensitivity in detecting the kidney changes induced by hyperuricemia.

In our study, diffusion-related parameters of renal cortex and medulla, such as ADC, D, and MD, had a significant decrease in hyperuricemia compared with those in healthy controls, indicative of restricted water molecule diffusion in the renal parenchyma. These parameters are all quantitative indexes of water molecule diffusion, and their common changing trends could prove each other although there are some subtle differences among them. As mentioned in our previous studies [12], an elevation in the serum urate concentration could trigger an inflammatory response, resulting in inflammatory cell proliferation and infiltration in renal parenchyma. Also, it could stimulate vascular smooth muscle cell proliferation and cause the deposition of urate crystal. These histopathological changes induced by hyperuricemia will reduce the intracellular or/and extracellular space and then limit the water molecule diffusion movement in renal parenchyma. Besides, with the renal tubular injury, interstitial fibrosis, and glomerular sclerosis occurring at the late stage of the disease, the diffusion of water molecules further declines. Moreover, we found that the D value was more valuable for differentiating AH from GA groups than other diffusion-related parameters (ADC and MD values), which is similar to some prior studies [9, 19, 20]. The reason is that D represents the pure diffusion, whereas MD and ADC values could also be affected by tubular fluid flow and capillary perfusion effects.

Furthermore, we found that the cortical and medullary FA values were lower in patients with hyperuricemia than those in the controls. FA is a common measurement used in DTI studies, which reflects the directionality of molecular displacement by diffusion. The decreasing FA value suggested the partly impaired renal microstructure. A similar finding was reported in some prior studies in diabetic nephropathy [21], renal allograft [22], sickle cell kidney disease [23] and CKD [9]. We speculated that those aforementioned factors, which contributed to the decreased diffusion-related parametric values, had the same effect in the FA value. Moreover, the pervious study [24] revealed that the direction of water molecule diffusion mainly depends on water molecule transport in tubules. High concentrations of uric acid may cause the reduction of water molecule flow rate in the tubule. This might be potential mechanisms for the decreased FA, as Lu et al. suggested in the diabetic nephropathy study [25]. In addition, urate crystal deposition in renal parenchyma could cause mild or even entire tubular congestion, which slows or prevents water molecule motion, resulting in the reduced FA value of renal parenchyma. This condition will be even more serious with the persistent state of high SUA, even the supersaturated condition (41/48).

We also observed a diminution of *f* and R2* values in the renal cortex and medulla in the HU group, compared with the HC group, whereas the significant differences were only discovered in the cortical R2* and medullary *f* values. In spite of their different algorithms, both of them could serve as the noninvasive indicators to monitor the changes in microscopic hemodynamics. The *f* value represents the ratio of blood vessels and tubular fluid volume to the total fluid in the tissue [20]. And the R2* value is a mirror of oxygen metabolism, which is inversely proportional to the content of oxygenated hemoglobin in tissues. Here, this seems to be a contradiction between the declined microscopic blood perfusion and higher oxygen content in the renal cortex and medulla in patients with hyperuricemia. However, this paradox might be a true and reasonable existence in kidneys because the renal haemodynamics and oxygenation are different from those of other organs, which has been mentioned in some previous studies [26–28]. Firstly, the kidney is the abundant blood organ which receives about 20% of the cardiac output under resting conditions. However, the blood perfusion within the kidneys is quite heterogeneous. 100% blood flow reaches the cortex, and only 15% of blood that previously passes through the cortex will reach the medulla. This unequal distribution contributes to the very low pO2 in the medulla [26]. Secondly, the kidney has a unique relationship between the metabolism and perfusion and the renal metabolism depends on its perfusion. When the renal blood perfusion increases, the glomerular filtration rate also enlarges and then the active transport processes of tubular resorption enhance to achieve the sodium balance. Tubular resorption requires an abundant of energy expenditure, which accounts for about 90% of the kidney’s energy expenditure, and, thus, a plenty of O_2_ is consumed [26]. Therefore, the decreased renal perfusion parameters could be accompanied by the increased oxygen content.

In addition, BOLD is based on a tight equilibrium assumption between the blood and tissue oxygenation and the R2* value is unable to display blood flow contribution alone. Thoralf et al. suggested that both renal blood oxygenation and physiological confounders with alterations in renal blood volume fraction (BVf) affected the renal T2*(1/R2*) to tissue pO2 relationship [26]. Meanwhile, this oxygen exchange between renal microvasculature and tissue could be affected by some factors, including fibrosis, anemia, and ischemia [29, 30]. This might be another cause of our result. Coincidentally, the analogically reduced D*, *f*, and R2* values in kidneys occurred in the GA group as well in comparison with the AH group, suggesting the rationality of these changes. Of course, considering the limited number of patients in this study, our results need, however, to be validated on a larger number of patients, probably in a multicenter study.

Interestingly, the current results demonstrated that five out of fourteen parameters had a capacity to further detect the differences between the subgroup of HU group, including cortical D, D^*^, and *f* values and medullary D and R2^*^ values. Then, the diagnostic performances of these five parameters were analyzed. It is worth noting that a combination of cortical D and *f* values and medullary R2* value demonstrated the highest AUC with the diagnostic efficacy of 0.900 and sensitivity and specificity of greater than 90%. This implies that a combination of different fMRI parameters is most sensitive in detecting the differences in renal function between patients with asymptomatic hyperuricemia and those with gouty arthritis and could be a promising biomarker for diagnosing the early renal damage in patients with hyperuricemia.

## Limitation

The primary limitation of this study was the absence of pathological confirmation about the fMRI parametric alterations because the invasive biopsy was not obtained from any patients with hyperuricemia included in this study. Our ongoing animal experiment will be a good surrogate for verifying the conclusion of this study. Another deficiency was the relatively small sample size. The future study with larger patient samples might provide more precise values and more convincing results.

## Conclusions

In conclusion, using multiparametric information is beneficial for us to comprehensively understand the alterations of the renal cortex and medulla affected by hyperuricemia from various perspectives. Moreover, a combination of different fMRI parameters could greatly improve the accuracy of the diagnosis and might be a promising tool for identifying early-stage renal injury.

## Supplementary Information

**Additional file 1**. The multiparametric maps of both kidneys

## Data Availability

All data generated or analysed during this study are included in this published article.

## Abbreviations

SUA: Serum uric acid
eGFR: Estimated glomerular filtration rate
IVIM: Intravoxel incoherent motion imaging
DTI: Diffusion tensor imaging
BOLD: Blood-oxygen-level-dependent
HC: Healthy control group
HU: Hyperuricemia group
AH: Asymptomatic hyperuricemia group
GA: Gouty arthritis group
AUC: Area under the receiver operating
ULT: Urate-lowering therapy

## Declarations

### Patient and Public Involvement

It was not appropriate or possible to involve patients or the public in the design, or conduct, or reporting, or dissemination plans of our research.
