# Supplementary material for: Multiparametric MRI analysis for the evaluation of renal function in patients with hyperuricemia: a preliminary study
Source: BMC Med Imaging. 2021 Sep 28;21:139. doi: 10.1186/s12880-021-00675-4 (PMC8477479; doi:10.1186/s12880-021-00675-4)
Supplement: Supplementary file 1 — Additional file 1. The multiparametric maps of both kidneys [file 12880_2021_675_MOESM1_ESM.docx]

**Supplementary materials**

| **Table 1: The ICCs of two observers on multiple parameters** | | | | | | | |
| --- | --- | --- | --- | --- | --- | --- | --- |
|  |  |  | Cortex | |  | Medulla | |
|  |  |  | ICC | 95% CI |  | ICC | 95% CI |
| **IVIM** | **ADC** |  | 0.922 | 0.874–0.951 |  | 0.883 | 0.811–0.927 |
|  | **D** |  | 0.935 | 0.895–0.959 |  | 0.961 | 0.937–0.976 |
|  | **D*** |  | 0.969 | 0.950–0.981 |  | 0.936 | 0.898–0.961 |
|  | ***f*** |  | 0.948 | 0.917–0.968 |  | 0.960 | 0.936–0.975 |
| **DTI** | **MD** |  | 0.846 | 0.752–0.904 |  | 0.878 | 0.804–0.924 |
|  | **FA** |  | 0.919 | 0.870–0.950 |  | 0.955 | 0.927–0.972 |
| **BOLD** | **R2*** |  | 0.952 | 0.922–0.970 |  | 0.971 | 0.953–0.982 |

ICC, intraclass correlation; CI, confidence intervals.

Supplemental Figure1


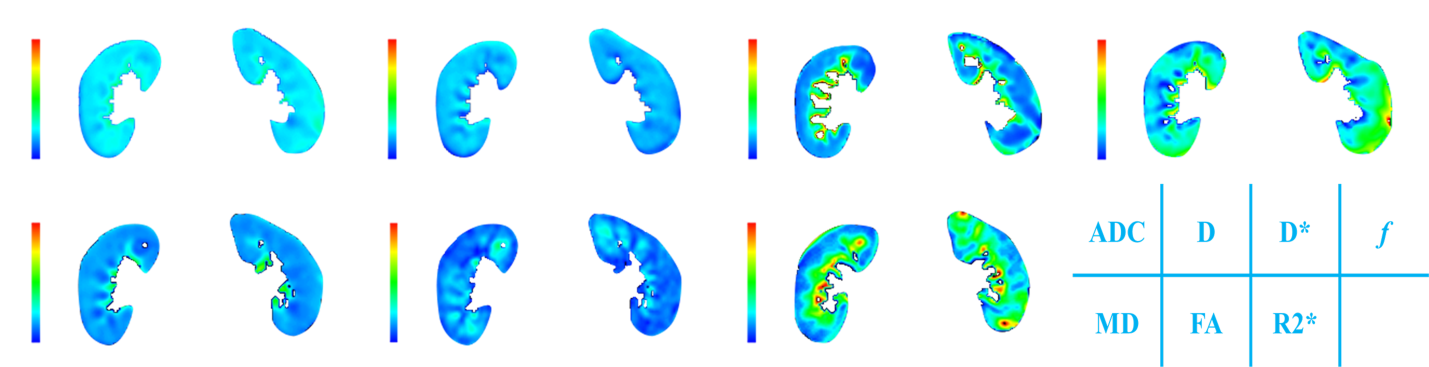


Example of multiparametric maps displays both kidneys in the coronal plane.

Supplemental Figure2


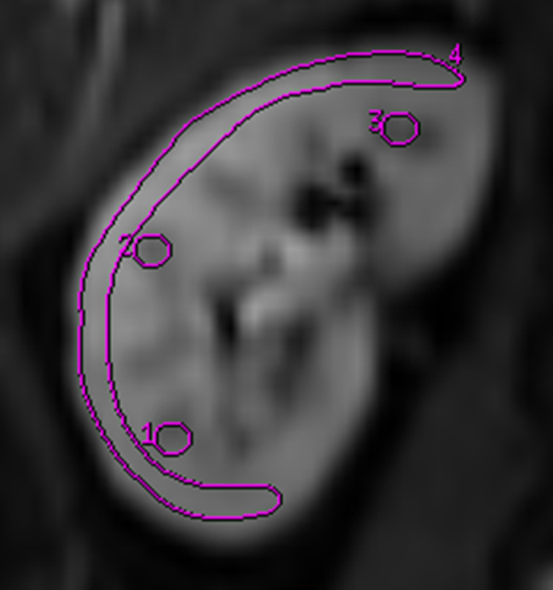


An example image shows the ROI placement of the renal cortex and medulla.
